# Travel-time barriers to specialized cancer care for adolescents and young adults with acute lymphoblastic leukemia

**DOI:** 10.1093/jncics/pkae046

**Published:** 2024-06-06

**Authors:** Helen M Parsons, Lori S Muffly, Ariadna Garcia, Amy Zhang, Kate Miller, David Van Riper, Kate Knowles, Theresa H Keegan

**Affiliations:** Division of Health Policy and Management, School of Public Health, University of Minnesota, Minneapolis, MN, USA; Division of Blood and Marrow Transplantation and Cellular Therapy, Stanford University, Stanford, CA, USA; Quantitative Sciences Unit, Stanford University, Stanford, CA, USA; Quantitative Sciences Unit, Stanford University, Stanford, CA, USA; Quantitative Sciences Unit, Stanford University, Stanford, CA, USA; Minnesota Population Center, University of Minnesota, Minneapolis, MN, USA; Minnesota Population Center, University of Minnesota, Minneapolis, MN, USA; Department of Internal Medicine, Division of Hematology/Oncology, University of California Davis School of Medicine, Sacramento, CA, USA

## Abstract

**Background:**

Prior studies demonstrate that 20%-50% of adolescents and young adults (age 15-39 years) with acute lymphoblastic leukemia (ALL) receive care at specialty cancer centers, yet a survival benefit has been observed for patients at these sites. Our objective was to identify patients at risk of severe geographic barriers to specialty cancer center–level care.

**Methods:**

We used data from the North American Association of Central Cancer Registries Cancer in North America database to identify adolescent and young adult ALL patients diagnosed between 2004 and 2016 across 43 US states. We calculated driving distance and travel time from counties where participants lived to the closest specialty cancer center sites. We then used multivariable logistic regression models to examine the relationship between sociodemographic characteristics of counties where adolescent and young adult ALL patients resided and the need to travel more than 1 hour to obtain care at a specialty cancer center.

**Results:**

Among 11 813 adolescent and young adult ALL patients, 43.4% were aged 25-39 years, 65.5% were male, 32.9% were Hispanic, and 28.7% had public insurance. We found 23.6% of adolescent and young adult ALL patients from 60.8% of included US counties would be required to travel more than 1 hour one way to access a specialty cancer center. Multivariable models demonstrate that patients living in counties that are nonmetropolitan, with lower levels of educational attainment, with higher income inequality, with lower internet access, located in primary care physician shortage areas, and with fewer hospitals providing chemotherapy services are more likely to travel more than 1 hour to access a specialty cancer center.

**Conclusions:**

Substantial travel-related barriers exist to accessing care at specialty cancer centers across the United States, particularly for patients living in areas with greater concentrations of historically marginalized communities.

Acute lymphoblastic leukemia (ALL) is a relatively rare, yet potentially curable, cancer that preferentially affects children and younger adults (1). Although 90% of newly diagnosed children with ALL in the United States achieve long-term survival, this estimate falls sharply to 40%-60% among newly diagnosed adolescents and young adults (aged 15-39 years) (2). Although the vast majority of children with ALL in the United States receives their ALL care in specialized cancer centers [defined as National Cancer Institute (NCI)–designated cancer centers (NCIDCC) (3) and/or Children’s Oncology Group (COG) sites (4)], only 20%-50% of adolescent and young adult ALL patients received frontline treatment at specialty cancer centers (5,6). Previous work has demonstrated that the proportion of adolescent and young adult ALL patients receiving frontline ALL treatment at specialty cancer centers declines steeply after the age of 17 years (5,7). Strikingly, studies have shown that 36% of adolescents and young adults receive frontline ALL therapy in facilities that treat no more than 1 adolescent and young adult ALL patient per year (8), highlighting the substantial diffusion of care for these complex patients. Receipt of frontline ALL treatment at a specialty cancer center is particularly important, as treatment at specialty cancer centers has been shown to result in a higher likelihood of receiving recommended pediatric-inspired ALL regimens (8), lower early mortality (7,9-11), and a higher likelihood of clinical trial enrollment and transplantation (12,13).

Geography plays a key role in accessing cancer care, as NCIDCC and COG facilities are not equally distributed across the United States, with fewer centers located in the central and mountain regions (3,4). Previously, we have shown that travel distance is associated with likelihood of receiving specialty care in California and that adolescents and young adults are independently more likely to receive treatment at a specialty cancer center if they are located within 10 miles of the center (14). Studies in other cancer populations have shown that patients who live farther from treatment facilities are not only more likely to be diagnosed at a later stage (15-17) but also less likely to complete recommended cancer treatments (16,18,19) and more likely to experience poorer survival (16,20,21). However, the majority of these studies were conducted in single states and focus on older populations with cancer (16). Further, these studies predominately focused on the individual characteristics of patients experiencing geographic or travel time–related barriers to cancer care rather than the communities in which they live, which can provide key insights into policy-relevant solutions for improving cancer care access.

As improving access to specialty cancer centers can play an important role in improving survival in adolescent and young adult ALL patients, we conducted a nationally representative, population-based analysis describing the geographic distribution of US adolescent and young adult ALL patients relative to specialty cancer centers and identifying patients at risk of severe geographical barriers to specialty cancer center–level care. As the association between NCI Community Oncology Research Program (NCORP) sites and adolescent and young adult ALL patient outcomes have not, to our knowledge, been assessed, we also explored NCORP sites, as the NCORP program may offer an opportunity for clinical trial participation (22).

## Methods

### Data

**Figure 1.**
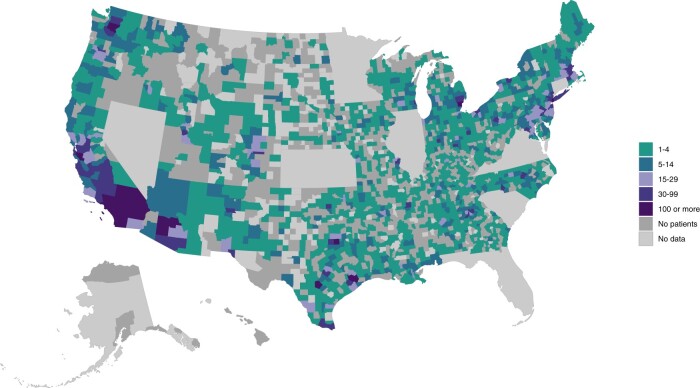
Geographic location of adolescent and young adult acute lymphoblastic leukemia patients in the United States diagnosed between 2004 and 2016 (n = 11 813) reported to the North American Association of Central Cancer Registries Cancer in North America database.

**Figure 2.**
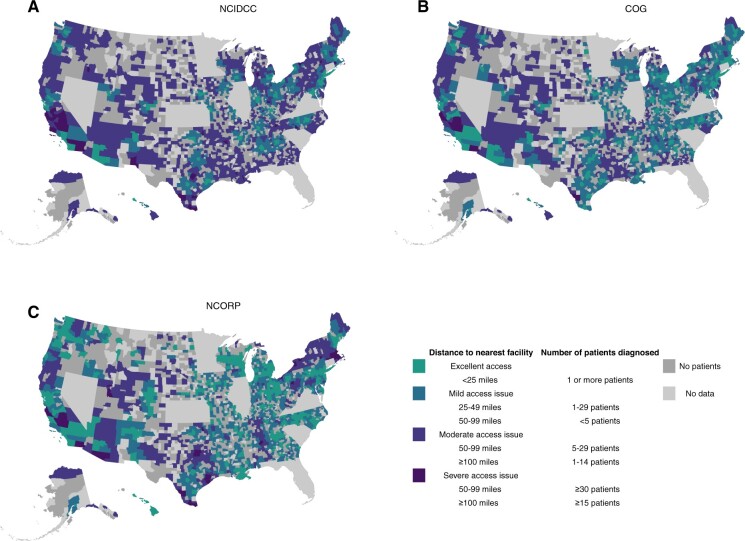
Geographic access among newly diagnosed adolescent and young adult acute lymphoblastic leukemia across the United States based on a composite of miles to the nearest facility and number diagnosed within that county, depicted separately for (**A**) National Cancer Institute Designated Cancer Centers, (**B**) Children’s Oncology Group sites, and (**C**) National Cancer Institute Community Oncology Research Program sites. COG = Children’s Oncology Group; NCI = National Cancer Institute; NCORP = NCI Community Oncology Research Program; NIDCC = NCI–designated cancer centers.

We used the Cancer in North America (CiNA) research data from the North American Association of Central Cancer Registries (NAACCR) for our study (23). These data include all Gold and Silver NAACCR-certified cancer registries participating in the National Cancer Institute’s Surveillance, Epidemiology, and End Results Program and the Center for Disease Control’s National Program of Cancer Registries (24). As cancer is a reportable disease, the participating registries collect information on each reported incident cancer case within their catchment areas, including demographics (eg, age at diagnosis, sex, and race and ethnicity), date of cancer diagnosis (month and year), tumor characteristics (eg, histology, grade, and stage), first course of therapy, and with permission, county at diagnosis. Each project requires individual cancer registry approval for release of their data for research. For this project, 43 states approved use of their data, with 7 states abstaining (Virginia, South Carolina, Florida, Minnesota, Illinois, Nevada, Kansas; light gray in Figures 1 and 2).

### Population

We included adolescents and young adults diagnosed between the ages of 15 and 39 years with ALL from 2004 to 2016 in the NAACCR CiNA research data who had information on residential county at diagnosis. Patients with duplicate entries and those living in Puerto Rico were excluded (Supplementary Figure 1, available online).

### Outcome measures

Our primary outcome was a travel time of at least 1 hour one way from the 2020 population-weighted county centroid to the nearest specialty cancer center, a common measure of excess travel distance to measure geographic barriers to care (25-27). To calculate travel time, we used the ArcMap 10.8.2 OD Cost Matrix tool from Network Analyst with the 2021 US Routing Network from ESRI Business. The routes were calculated to optimize driving distance and restricted to driving an automobile with no prioritization of highways or avoiding tolls (see Supplementary Methods, available online for additional details).

We additionally wanted to evaluate the relative burden of an adolescent and young adult diagnosis for specific counties that accounted for not only the travel burden but also the population impacted. As no established measure exists, we created a composite measure of population-based geographic access using a combination of information on the travel distance required to get to the closest specialty cancer center and the number of patients diagnosed in a county during the study period, assigning each county as having excellent access, mild access issues, moderate access issues, or severe access issues (see Supplementary Methods, available online) to describe the relative burden to access specialty cancer centers by county. The goal of creating this composite measure was not to evaluate the validity of this measure or association with our primary outcome but rather to highlight areas where there are more patients and longer drive times. The cut-points of the number of patients are mutually exclusive within distance to facility categories; however, the distribution of patients by distance to facility varied such that there are different categories for patient volume within distance categories. Geographic access to an NCIDCC, COG, or NCORP site was then mapped by county.

### Predictors of travel time to an specialty cancer center among adult and young adolescent ALL patients

To understand factors that may influence geographic access to care for adolescents and young adults with ALL, we used a conceptual model based on the behavioral-ecological framework of health-care access and navigation (28). In this model, there are a set of key driving factors that directly influence health behaviors (eg, the distance someone must travel to access a specialty cancer center and ultimately choosing treatment at a specialty cancer center), which will then influence their overall outcomes, particularly survival. We used this model to select county-level factors that could impact access to care in our population, including the characteristics of the communities in which adolescents and young adults diagnosed with ALL live, their social environment, their built environment, and the health-care environment of their community. County-level measures were chosen to characterize the community-level factors that most influence access to specialty cancer centers to identify policy-relevant factors for potential intervention. Using information from the Agency for Healthcare Research and Quality Social Determinates of Health database (29), which creates a single source for social determinant of health variables by aggregating data across multiple source files (eg, American Community Survey [ACS], County Health Rankings), we linked county-level attributes for counties where at least 1 adolescent and young adult patient with ALL was diagnosed during our study period. Measures of county-level demographics included geographic region (30) (Midwest, Northeast, Southeast, Southwest, West), whether a county was located in a metropolitan vs nonmetropolitan area on the basis of the 2013 rural-urban continuum codes (RUCC) (31) of the patient’s residence at diagnosis in CiNA (RUCC 1-3 were coded as metropolitan; RUCC 4-9 nonmetro; variable: Area Health Resource file [AHRF]_USDA_RUCC_2013) and the percentage of the county with a bachelor’s degree (in tertiles; variable: ACS_PCT_BACHELOR_DGR) based on the ACS 5-year estimates (2012-2016) (32). Measures of the county-level social environment included income inequality (in tertiles), measured by the GINI index (33) from the 2012-2016 ACS 5-year estimates (variable: ACS_GINI_INDEX) and segregation, measured by the 2018 County Health Rankings residential segregation index (Index of Dissimilarity) (34) indicating greater residential segregation between Black and White residents (variable: CHR_SEGREG_BLACK). Measures of the county-level built environment included no vehicle ownership (variable: ACS_PCT_HU_NO_VEH) and no internet access (variable; ACS_PCT_HH_NO_INTERNET), measured as the county-level percentage of the population with no vehicle and no internet access (in tertiles), measured from the 2012-2016 and 2013-2017 5-year ACS estimates, respectively, as 5-year estimates for internet use were not available for earlier time periods. Finally, we examined the county-level health-care environment by evaluating whether the county (in part or as a whole) was part of a primary care provider shortage area (variable: AHRF_HPSA_PRIM) and the number of emergency medicine physicians in the county per 1000 population (in tertiles; variable: AHRF_ER_MED_RATE) using data from the 2019-2020 and 2017-2018 AHRF (35), respectively. The number of hospitals offering chemotherapy in the county was based on the 2016 Provider of Services (36) file (variable: POS_TOT_HOSP_CHEMO). As we found little variation in assigned county-level characteristics based on our choice of measure year (ie, when we examined the values for the same measures, when available, across our study period), we selected the county characteristics closest to the end of study period for analysis. Source documentation for all variables used in the analysis can be found on the Agency for Healthcare Research and Quality Social Determinants of Health database website (29).

### Statistical analysis

We first conducted descriptive analyses to examine the patient demographics and county-level attributes of individuals diagnosed with adolescent and young adult ALL. Our multivariable analysis focused on county-level attributes that all individuals living in that county may be exposed to rather than their individual characteristics to examine factors that may inform policy-relevant solutions for improving cancer care access. We used multivariable logistic regression models to examine the relationship between characteristics of counties where adolescents and young adults with ALL resided and the need to travel more than 1 hour one way to obtain care at a 1) NCIDCC, 2) COG facility, or 3) any specialty cancer center (NCIDCC or COG). We evaluated correlation between variables within each domain (eg, social environment) included in our final models using Spearman rank correlation coefficient and found low levels of correlation (<0.3) for all variables. We conducted sensitivity analyses removing Alaska and Hawaii from analyses to explore whether our results remained consistent excluding these states as travel time was calculated differently for these areas (see Supplementary Methods, available online). As NCORP analyses were exploratory, we only described distance and access issues from NCORP sites and did not include NCORP sites in the models. For all models, 2-sided *P* values less than .05 were considered statistically significant. All analyses were performed using SAS version 9.3 (Cary, NC, USA). This study was approved by the institutional review boards of Stanford University.

## Results

### Patient characteristics and geographic location

We identified 11 813 adolescent and young adult ALL patients diagnosed between 2004 and 2016 in 43 states across the United States. The majority were young adults (aged 20-39 years; 64.3%), male (65.5%), and either non-Hispanic White (51.5%) or Hispanic (32.9%) (Table 1). Of the patients, 48.9% were privately insured, with 28.7% covered by public insurance and 7.4% uninsured.

**Table 1.** Individual and county-level characteristics of adolescent and young adult acute lymphoblastic leukemia patients diagnosed between 2004 and 2016 reported to the North American Association of Central Cancer Registries Cancer in North America database, stratified by traveling time to a specialized cancer center

Characteristics	Total patients, No. (%)	Less than 1-hour traveling time to reach a specialty cancer center, No. (%)	At least 1-hour traveling time to reach a specialty cancer center, No. (%)	*P*
	(n = 11813)	(n = 9023)	(n = 2790)	
Individual-level characteristics				
Age at diagnosis, y				.001
15-19	4213 (35.7)	3138 (34.8)	1075 (38.5)	
20-24	2467 (20.9)	1871 (20.7)	596 (21.4)	
25-29	1893 (16.0)	1485 (16.5)	408 (14.6)	
30-34	1593 (13.5)	1259 (14.0)	334 (12.0)	
35-39	1647 (13.9)	1270 (14.1)	377 (13.5)	
Sex				.544
Male	7739 (65.5)	5925 (65.7)	1814 (65.0)	
Female	4074 (34.5)	3098 (34.3)	976 (35.0)	
Insurance at diagnosis				.058
Military	39 (0.3)	34 (0.4)	5 (0.2)	
Private	5775 (48.9)	4412 (48.9)	1363 (48.9)	
Public	3386 (28.7)	2544 (28.2)	842 (30.2)	
Uninsured	874 (7.4)	671 (7.4)	203 (7.3)	
Unknown	1739 (14.7)	1362 (15.1)	377 (13.5)	
Race and ethnicity				<.001
Hispanic, all races	3886 (32.9)	3267 (36.2)	619 (22.2)	
Non-Hispanic Black	1001 (8.5)	809 (9.0)	192 (6.9)	
Non-Hispanic White	6079 (51.5)	4227 (46.8)	1852 (66.4)	
Other or unknown^a^	847 (7.2)	720 (8.0)	127 (4.6)	
County-level characteristics^b^				
Geographic region				<.001
Midwest	1905 (16.1)	1345 (14.9)	560 (20.1)	
Northeast	2583 (21.9)	2196 (24.3)	387 (13.9)	
Southeast	1802 (15.3)	1012 (11.2)	790 (28.3)	
Southwest	1975 (16.7)	1621 (18.0)	354 (12.7)	
West	3548 (30.0)	2849 (31.6)	699 (25.1)	
Metropolitan residence				<.001
Nonmetro	1482 (12.5)	276 (3.1)	1206 (43.2)	
Metro	10 331 (87.5)	8747 (96.9)	1584 (56.8)	
% of population with at least a bachelor’s degree				<.001
2.34-16.03, lowest tertile	3948 (33.4)	1991 (22.1)	1957 (70.1)	
16.04-20.68, middle tertile	4024 (34.1)	3527 (39.1)	497 (17.8)	
20.69-43.04, highest tertile	3841 (32.5)	3505 (38.8)	336 (12.0)	
GINI index of income inequality				<.001
0.35-0.45, lowest tertile	4711 (39.9)	3143 (34.8)	1568 (56.2)	
0.46-0.47, middle tertile	3217 (27.2)	2437 (27.0)	780 (28.0)	
0.48-0.6, highest tertile	3885 (32.9)	3443 (38.2)	442 (15.8)	
Residential segregation index				<.001
0.57-47, lowest tertile	3863 (32.7)	2900 (32.1)	963 (34.5)	
47.01-57.85, middle tertile	4311 (36.5)	3570 (39.6)	741 (26.6)	
57.86-90.37, highest tertile	3205 (27.1)	2473 (27.4)	732 (26.2)	
Unknown	434 (3.7)	80 (0.9)	354 (12.7)	
Percentage of housing units with no vehicle available				<.001
0.65-5.8, lowest tertile	3951 (33.4)	2844 (31.5)	1107 (39.7)	
5.81-8.32, middle tertile	3937 (33.3)	2776 (30.8)	1161 (41.6)	
8.33-77.26, highest tertile	3925 (33.2)	3403 (37.7)	522 (18.7)	
Designated health profession shortage area (primary care)				<.001
0, no shortage	867 (7.3)	612 (6.8)	255 (9.1)	
1, whole county	473 (4.0)	130 (1.4)	343 (12.3)	
2, partial county	10 473 (88.7)	8281 (91.8)	2192 (78.6)	
No. of nonfederal emergency medicine physicians in a county per 1000 population				<.001
0-0.08, lowest tertile	4027 (34.1)	2192 (24.3)	1835 (65.8)	
0.09-0.14, middle tertile	3907 (33.1)	3353 (37.2)	554 (19.9)	
0.15-0.75, highest tertile	3879 (32.8)	3478 (38.5)	401 (14.4)	
Total number of hospitals offering chemotherapy				<.001
≤1, lowest tertile	3949 (33.4)	1896 (21.0)	2053 (73.6)	
>1 to 6, middle tertile	2226 (18.8)	1589 (17.6)	637 (22.8)	
>6, highest tertile	5638 (47.7)	5538 (61.4)	100 (3.6)	
Percentage of households with no internet access				<.001
3.36-14.2, lowest tertile	4020 (34.0)	3573 (39.6)	447 (16.0)	
14.21-18.79, middle tertile	3863 (32.7)	3359 (37.2)	504 (18.1)	
18.8-71.04, highest tertile	3930 (33.3)	2091 (23.2)	1839 (65.9)	

a Other or unknown includes all other or unknown races and ethnicities.

b Based on the individual’s county of residence at diagnosis.

Figure 1 depicts the geographic location of all newly diagnosed adolescent and young adult ALL patients in the United States (excluding the 7 states with no data provided). Regionally, patients were most often diagnosed in the West (30.0%) and Northeast (21.9%); however, the states with the greatest numbers of newly diagnosed adolescent and young adult ALL patients were Texas, California, New York, Pennsylvania, and Georgia (Supplementary Table 1, available online). At least 1 adolescent and young adult ALL patient was diagnosed in a total of 1537 unique US counties (59.4% of the 2586 counties included in this study). Among these, 69% (n = 1073) of counties had less than 5 patients diagnosed during the study period, 20% of counties with 5-14 patients diagnosed, and 5% each with 15-29 patients and at least 30 patients diagnosed (Supplementary Table 2, available online).

### Geographic access to a specialty cancer center

**Figure 3.**
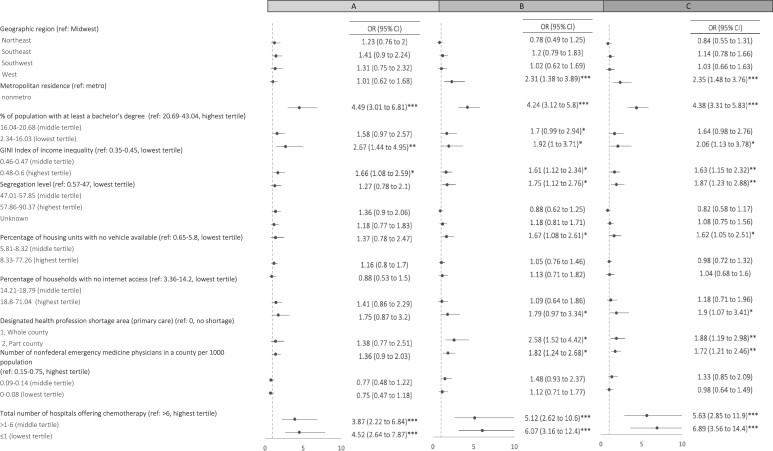
Multivariable logistic regression models evaluating county-level variables associated with driving time of more than 1 hour each way to a (**A**) National Cancer Institute Designated Cancer Center, (**B**) Children’s Oncology Group site, and (**C**) specialized cancer center (n = 1537 counties). Models include all counties where adolescents and young adults with acute lymphoblastic leukemia were diagnosed within the North American Association of Central Cancer Registries Cancer in North America database. Each model is adjusted for geographic region and county-level measures of metropolitan residence, education, income inequality, segregation, vehicle ownership, internet access, primary care provider shortage areas, and the per capita number of emergency medicine physicians and hospitals providing chemotherapy. Values represent the *P* value of the point estimate relative to the reference group for each variable in the model. Statistically significant values specified as *P < .05, **P < .01, ***P < .001. CI = confidence interval; OR = odds ratio; ref = referent.

We found that 2790 (23.6%; Table 1) of adolescent and young adult ALL patients from 60.8% (n = 936) of included US counties would be required to travel at least 1 hour one way to access a specialty cancer center. Geographic access requiring travel of at least 1 hour one way affected more patients (5005 [42%] vs 2821 [24%]) and more counties (1245 [81%] vs 942 [61%]) when evaluating travel distance from an NCIDCC compared with a COG site, respectively (data not shown). Patients living in nonmetro counties, counties with lower levels of educational attainment, those with higher income inequality, and those with fewer hospitals providing chemotherapy were more likely to require travel at least 1 hour one way to access an NCIDCC (Figure 3, A; *P* < .05 for all). Patients living in counties in the western United States, in nonmetro counties, counties with lower levels of educational attainment, areas with higher income inequality, in a professional shortage area, and fewer hospitals providing chemotherapy were more likely to require travel at least 1 hour one way to access a COG site (Figure 3, B). Combined models examining travel time of at least 1 hour to any specialty cancer center showed similar results while identifying lower levels of internet access as a statistically significant factor requiring longer travel time (Figure 3, C). Our sensitivity analyses demonstrated consistent results when Alaska and Hawaii were excluded from our analyses.

We combined adolescent and young adult ALL patient density and travel distance to create a new county-level variable signifying severity of specialty cancer center access. Using this composite variable, we found that 5271 (45%) patients would have excellent access to an NCIDCC, whereas 4725 (40%) patients would have a moderate or severe issue accessing an NCIDCC (Figure 2, A; Supplementary Table 2, available online). In terms of accessing a COG site, 1036 (9%) patients would have excellent access to a COG site, whereas 3621 (30%) patients would have a severe issue accessing a COG site (Figure 2, B; Supplementary Table 2, available online).

### NCI Community Oncology Research Program

We explored travel distance to NCORP sites among newly diagnosed adolescent and young adult ALL patients across the United States. We found that 1613 (14%) patients of newly diagnosed adolescent and young adult ALL patients would have excellent access to an NCORP site, whereas 3309 (28%) patients would have a severe geographical access issue (Figure 2, C; Supplementary Table 2, available online).

## Discussion

In our population-based study of adolescents and young adults diagnosed with ALL across the United States, we found that almost one quarter of patients living in more than 60% of included counties experience substantial geographic barriers (ie, >1 hour travel time each way) to accessing specialty cancer centers, either through an NCIDCC or COG site. Importantly, patients living in areas with greater concentrations of historically marginalized communities, such as counties with greater income inequality and lower levels of educational attainment, remain most at risk for travel time–related barriers to accessing these facilities. As prior work by our team has demonstrated that adolescents and young adults with ALL experienced superior survival following treatment at a specialty cancer center (9-11), our work can inform policies to enhance access to specialty cancer centers for those populations most at risk for geographic barriers.

ALL is a rare and complex disease. Although, historically, children with ALL have benefited from a relatively unified treatment approach because most children are treated at specialty cancer centers, including COG-designated centers (5,9,14,37,38), the treatment approach for adolescent and young adult ALL patients appears to be more consistent at centers with greater expertise (eg, specialty cancer centers and centers with higher adolescent and young adult ALL patient volumes) (5,39). From this perspective, treatment at a specialty cancer center for rare and complex cancers such as ALL is important, as these centers tend to have leukemia specialists, cellular therapy specialists, access to leukemia-specific clinical trials, associated infectious disease teams, and other ancillary teams helping provide care for acute leukemia patients (39). For adolescents predominately treated at pediatric centers, COG designation provides an analogous level of specialty care for pediatric leukemia, as these centers are considered centers of excellence for pediatric cancer care, have access to COG ALL trials, and have been vetted by COG as ALL specialists (40). Yet, there are currently only 71 NCIDCCs across the United States and just over 200 COG member institutions (3,4). Although NCORP centers appear more geographically accessible for adult patients relative to NCIDCCs, work is needed to understand whether these sites consistently provide comprehensive ALL care and ALL clinical trial options across the United States and, if so, identify ways to improve access, as recent analyses have suggested that few adolescents and young adults with ALL (<1% to 13%) are treated at NCORP facilities (7). Importantly, the location of specialty cancer centers are not equally distributed, with critical gaps in the West region of the United States. As a result, large segments of the population of adolescents and young adults with ALL require greater than 1-hour travel time each way from their county of residence if they were to obtain care at a specialty cancer center. Extended travel times may have negative consequences on a multitude of domains such as treatment compliance, caregiver burnout, and financial toxicity, as has been shown in other settings (18,25,41).

In our prior work, we have shown that where adolescents and young adults with ALL live is associated with the likelihood of receiving specialty care in California. Specifically, adolescents and young adults are independently more likely to receive treatment at a specialty cancer center if they are located within 10 miles of the center (14). Work in other populations with cancer have shown that those who live farther from treatment facilities are not only more likely to be diagnosed at a later stage (15-17) but also less likely to complete recommended cancer treatments (16,18,19) and more likely to experience poorer survival (16,20,21). For example, in a study of older women with breast cancer who underwent breast-conserving surgery, those living farther from radiation facilities were less likely to receive guideline-concordant radiation therapy after surgery (18). Although prior studies provide important context for the role of geographic access on cancer care and outcomes, much of this work has focused on some of the most common solid tumors (eg, breast, colon) where community oncologists have large volumes of patients and would likely feel comfortable treating individuals under standard guideline-recommended protocols. Because of its complex and rare nature, where more than 36% of adolescents and young adults receive frontline ALL therapy in facilities that treat no more than 1 adolescent and young adult ALL patient per year (8), travel distance can play an even more important role in accessing specialized care from providers with broad experience in treating these types of cancer. Importantly, our work highlights not only the distance adolescents and young adults with ALL must travel to receive care at a specialty cancer center but also the communities most impacted by these barriers. Specifically, we find that individuals living in nonmetropolitan counties as well as counties with greater income inequality, lower levels of educational attainment, and less access to internet remain most at risk for geographic barriers to accessing these facilities. We know from prior literature that rural residents, those with lower income levels (and often lower rates of being insured), and those with lower levels of educational attainment already put patients with ALL at risk of lower treatment adherence and poor outcomes (42-45). Geographic barriers remain a key additional barrier to these communities, recognizing that the majority of included counties in our study where adolescents and young adults with ALL live require more than 1-hour travel time to reach a specialty cancer center.

To address community-level factors that are associated with increased travel time to adolescent and young adult ALL patient care across the United States, policy makers and payers may look to state and regional initiatives aimed at increasing specialty cancer center access. Other specialties, such as pediatric oncology (37), have developed collaborative care networks for ALL management, and other countries (46) have consolidated care and prioritized clinical trials for adolescent and young adult ALL patients. Coverage for hematopoietic cell transplantation, a complex and expensive procedure for blood cancer patients, is often predicated on center outcomes and expertise (47). Recently, new legislation in California (Cancer Care Equity Act) would require Medi-Cal (ie, California Medicaid) managed care plans to make a good faith effort to contract with at least 1 specialty cancer center in its network (eg, NCIDCC, NCORP) and allow enrollees diagnosed with a complex cancer diagnosis to request a referral to those centers for medically necessary services (48). New York currently has a similar demonstration project (S7614) that would ensure individuals enrolled in Medicaid-managed care have greater access to an NCIDCC. Although initiatives such as these provide important first steps in improving access to specialty cancer center care by creating broader networks with the goal of reducing travel-time burdens, we also demonstrate the importance of considering the needs of the communities experiencing the greatest geographic barriers to specialty cancer center care, recognizing higher levels of income inequality, lower internet access, and educational attainment in these communities. Several innovative initiatives within health systems are focusing efforts in this space, connecting individuals to needed social and community services (eg, transportation, housing, food support) to address these needs (49). As the Centers for Medicare & Medicaid Services has released guidance for state to use Medicaid to address social determinants of health (50), states will likely have increased flexibility from Centers for Medicare & Medicaid Services in addressing the needs of the most vulnerable adolescents and young adults with ALL to ensure they have broad access to specialty cancer centers, particularly in communities with higher travel-related burdens. This guidance can serve as a starting point for initiating innovative policies that can directly address access to specialized centers for individuals living in underresourced communities far from these centers. For example, some Medicaid agencies reimburse members for ancillary travel expenses (eg, lodging, meals, parking, and tolls) necessary to obtain covered health services (51). Other innovative approaches include the Medicaid-for-groceries initiative, a test program that allows states to spend funds on health-related needs such as nutrition counseling and healthy-meal preparation (52), as well as housing stabilization services to help individuals on Medicaid find and keep secure housing (53). Expanding these benefits beyond state-based Medicaid initiatives to include other states and payers presents opportunities to address the needs of individuals in their communities and improve access to timely, recommended care within specialized centers.

Our population-based study presents the first, to our knowledge, national assessment of geographic access barriers for young adults with ALL; however, we recognize certain limitations. First, on the basis of available data from the NAACCR registries, the CiNA data only releases county of residence for individuals in the cancer registry. As a result, we made certain assumptions about the average travel distance individuals living in a county would have to travel to access a specialty cancer center, using population-weighted centroids to account population distributions, which allow us to estimate the average driving time someone living in a particular county would be required to travel to reach the nearest specialized cancer center. Additionally, a small number (n = 7) of state registries did not release data for this study. Our study does, however, include a geographically and demographically diverse segment of the majority of the US population of adolescents and young adults with ALL identifying areas at highest risk for geographic barriers and opportunities for policy intervention within communities. We additionally acknowledge the lack of temporal alignment for our community-level measures in our study, which varied in availability across our study period. We do note, however, consistency of values within these measures during our study years. Finally, we acknowledge that the characteristics of the individual’s county of residence may not apply to all of the ALL patients residing in that county, which is a limitation of the ecologic study design. Future studies should examine these associations at the patient level. However, this can serve as a starting point for additional context in understanding characteristics of communities who experience time travel–related burdens to accessing specialty cancer centers.

In conclusion, we demonstrate a broad, national assessment of geographical opportunities and limitations in accessing specialty cancer centers among adolescent and young adult patients with ALL. Our work highlights the communities most impacted by travel time–related barriers, including individuals living in nonmetropolitan counties as well as counties with greater income inequality and lower levels of educational attainment, that put patients with ALL at risk of lower treatment adherence and poor outcomes. Given the importance of specialty cancer center care on outcomes for this population, a multipronged approach to addressing barriers to care such as collaborative models of care or new mechanisms to identify and certify programs for ALL expertise is necessary to address geographical constraints.

## Supplementary Material

pkae046_Supplementary_Data

## Data Availability

The study statistical analysis plan will be made available upon request to lmuffly@stanford.edu. The data that support the findings of this study are available from the North American Association of Central Cancer Registries (NAACCR). Access is granted through an application process to each registry by the management or data custodians.
