# Supplementary material for: Travel-time barriers to specialized cancer care for adolescents and young adults with acute lymphoblastic leukemia
Source: JNCI Cancer Spectr. 2024 Jun 6;8(4):pkae046. doi: 10.1093/jncics/pkae046 (PMC11229987; doi:10.1093/jncics/pkae046)
Supplement: pkae046_Supplementary_Data [file pkae046_supplementary_data.pdf]

Supplementary Material

Supplementary Methods

Additional Details on Study Measures

Distance to Specialized Cancer Centers (SCCs)

Our primary outcome was a travel time ≥1 hour one-way from the 2020 population-weighted county centroid to the nearest SCC, a common measure of excess travel distance to measure geographic barriers to care. [1-3] To measure this, we obtained county at diagnosis (the smallest geographic unit of analysis released by NAACCR) for each individual. We then identified the addresses of all NCIDCC,[4] COG[5] and NCORP[6] sites from publicly available websites. Travel time from county of residence to the closest SCC was calculated using the population-weighted centroid for a participant's county of residence (using 2020 decennial census data) and the geocoded location of the closest NCIDCC, COG and NCORP site (separately) using ArcGIS.[7]

To do this, we geocoded all treatment facilities in ArcGIS Pro 3.1.0 with the Local Composite geocoder. To calculate driving distance and time, we used ArcMap 10.8.2. We used the OD Cost Matrix tool from Network Analyst with the 2021 US Routing Network from Esri Business Analyst. The routes calculated were optimized for driving distance and restricted to driving an automobile with no prioritization of highways or avoiding tolls. For Hawaii and Alaska only, straight line distances were used because patients and facilities are separated by water, separated by another country or the counties are so large that county population-weighted centroids had to be moved long distances to attach to the road network. Straight-line distances were calculated using the Generate Near Tool in ArcGIS Pro 3.1.0.

We chose 2020 population-based centroids in order to incorporate the most recently available population data. For the majority of states, the median distance between the 2010 and 2020 population-weighted centroids was less than 400 meters, a relatively small difference in driving distance.

Geographic Access to SCCs

We created a composite measure of population-based geographic access using a combination of information on the travel distance required to get to the closest SCC and the number of patients diagnosed in a county during the study period, assigning each county as having excellent access (<25 miles from SCC and 1 or more patients diagnosed in the county), mild access issues (25-49 miles from SCC and 1 or more patients diagnosed OR 50-99 miles from SCC but <5 patients diagnosed), moderate access issues (50-99 miles from SCC and 5-29 patients diagnosed OR >=100 miles from SCC and 1-14 patients diagnosed), or severe access issues (50-99 miles from SCC and >=30 patients diagnosed OR >=100 miles from SCC and >=15 patients diagnosed)

References

1. Rocque GB, Williams CP, Miller HD, et al. Impact of Travel Time on Health Care Costs and Resource Use by Phase of Care for Older Patients With Cancer. J Clin Oncol 2019;37:1935-45.
2. Scoggins JF, Fedorenko CR, Donahue SM, Buchwald D, Blough DK, Ramsey SD. Is distance to provider a barrier to care for medicaid patients with breast, colorectal, or lung cancer? J Rural Health 2012;28:54-62.
3. Onega T, Duell EJ, Shi X, Wang D, Demidenko E, Goodman D. Geographic access to cancer care in the U.S. Cancer 2008;112:909-18.
4. NCI-Designated Cancer Centers. 2023. (Accessed February 12, 2023, at https://www.cancer.gov/research/infrastructure/cancer-centers.)
5. Children's Oncology Group Locations. 2023. (Accessed February 22, 2023, at https://childrensoncologygroup.org/locations.)
6. Community Oncology Research Program: NCORP Sites. 2023. (Accessed May 1, 2023, at https://ncorp.cancer.gov/findasite/.)
7. ArcGIS 2023. (Accessed June 16, 2023, at https://www.arcgis.com/index.html.)

Supplementary Table 1. Number of Adolescent and Young Adults with Acute Lymphoblastic Leukemia by state reported to the North American Association of Central Cancer Registries (NAACCR CiNA database, 2004-2016

State	n (%) N = 11,813
Alabama	161 (1.4)
Alaska	28 (0.2)
Arizona	347 (2.9)
Arkansas	108 (0.9)
California	2,344 (20)
Colorado	207 (1.8)
Connecticut	152 (1.3)
Delaware	41 (0.3)
District of Columbia	30 (0.3)
Georgia	430 (3.6)
Hawaii	45 (0.4)
Idaho	78 (0.7)
Indiana	249 (2.1)
Iowa	124 (1.0)
Kentucky	141 (1.2)
Louisiana	161 (1.4)
Maine	38 (0.3)
Maryland	195 (1.7)
Massachusetts	262 (2.2)
Michigan	534 (4.5)
Mississippi	91 (0.8)
Missouri	190 (1.6)
Montana	35 (0.3)
Nebraska	87 (0.7)
New Hampshire	58 (0.5)
New Jersey	376 (3.2)
New Mexico	117 (1.0)
New York	881 (7.5)
North Carolina	366 (3.1)
North Dakota	24 (0.2)
Ohio	427 (3.6)
Oklahoma	151 (1.3)

Oregon	180 (1.5)
Pennsylvania	551 (4.7)
Rhode Island	41 (0.3)
South Dakota	35 (0.3)
Tennessee	227 (1.9)
Texas	1,360 (12)
Utah	128 (1.1)
Vermont	29 (0.2)
Washington	483 (4.1)
West Virginia	46 (0.4)
Wisconsin	235 (2.0)
Wyoming	20 (0.2)

Supplementary Table 2. Number of Counties by One-Way Distance to Specialized Cancer Center and Number of Adolescent and Young Adult Acute Lymphoblastic Leukemia patients diagnosed in that County during the study period (N=1,537 Counties)

Excellent access | Mild access issue | Moderate access | Severe access

Distance to a National Cancer Institute Designated Cancer Center (n, %)

	One-Way Distance to a Specialized Cancer Center			
	0 - 24 mi	25 - 49 mi	50 - 99 mi	>= 100 mi
Patients*				
<5	24 (2)	102 (7)	324 (21)	623 (41)
5 to 14 pats	28 (2)	59 (4)	89 (6)	131 (9)
15 to 29 pats	27 (2)	21 (1)	15 (1)	16 (1)
>=30	53 (3)	11 (1)	8 (1)	6 (0)
**Total Counties**	132 (9)	193 (13)	436 (29)	776 (51)
**Total Patients**	5,271 (45)	1,817 (15)	2,007 (17)	2,718 (23)

* Number Diagnosed in the County During the Study Period

Distance to a Children's Oncology Group Facility (n, %)

	One-Way Distance to a Specialized Cancer Center			
	0 - 24 mi	25 - 49 mi	50 - 99 mi	>= 100 mi
Patients*				
<5	70 (5)	260 (17)	429 (28)	314 (20)
5 to 14 pats	81 (5)	92 (6)	77 (5)	57 (4)
15 to 29 pats	49 (3)	20 (1)	8 (1)	2 (0)
>=30	67 (4)	8 (1)	2 (0)	1 (0)
**Total Counties**	267 (17)	380 (25)	516 (34)	374 (24)
**Total Patients**	1,036 (9)	7,156 (61)	2,052 (17)	1,569 (13)

* Number Diagnosed in the County During the Study Period

Distance to an National Cancer Institute Community Oncology Research Program Facility (n, %)

	One-Way Distance to a Specialized Cancer Center			
	0 - 24 mi	25 - 49 mi	50 - 99 mi	>= 100 mi
Patients*				
<5	245 (16)	279 (18)	328 (21)	221 (14)
5 to 14 pats	152 (10)	48 (3)	55 (4)	52 (3)
15 to 29 pats	43 (3)	11 (1)	10 (1)	15 (1)
>=30	52 (3)	5 (0)	12 (1)	9 (1)
**Total Counties**	492 (32)	343 (22)	405 (27)	297 (19)
**Total Patients**	1,613 (14)	6,891 (58)	1,398 (12)	1,911 (16)

* Number Diagnosed in the County During the Study Period

Supplementary Figure 1. Study Flow Diagram Applying Inclusion and Exclusion Criteria

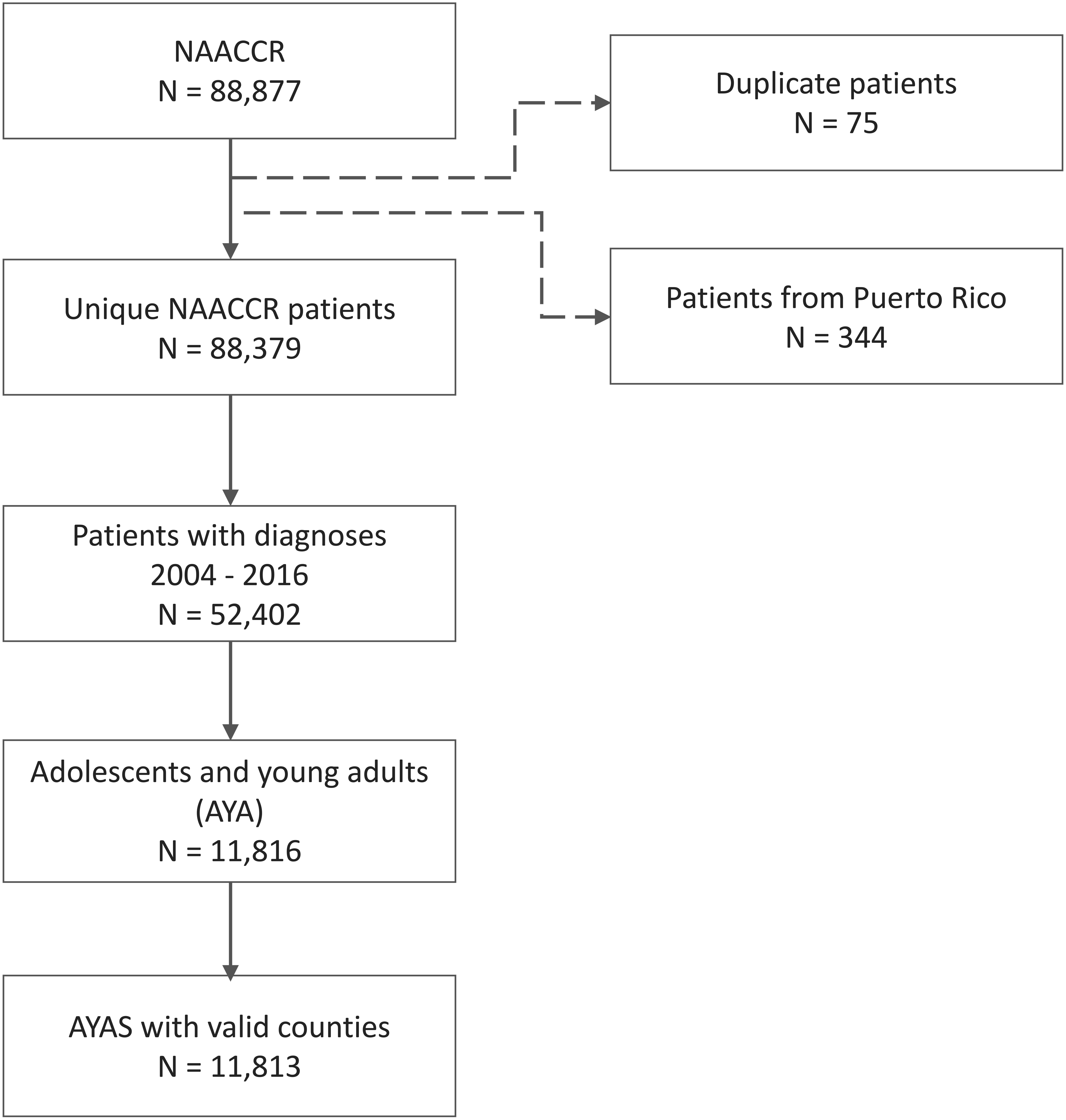
NAACCR
N = 88,877
Duplicate patients
N = 75
Patients from Puerto Rico
N = 344
Unique NAACCR patients
N = 88,379
Patients with diagnoses
2004 - 2016
N = 52,402
Adolescents and young adults
(AYA)
N = 11,816
AYAS with valid counties
N = 11,813
